# Supplementary material for: Growth Arrest of Alveolar Cells in Response to Cytokines from Spike S1-Activated Macrophages: Role of IFN-γ
Source: Biomedicines. 2022 Dec 1;10(12):3085. doi: 10.3390/biomedicines10123085 (PMC9775973; doi:10.3390/biomedicines10123085)
Supplement: Supplementary file 1 [file biomedicines-10-03085-s001.zip › biomedicines-2028779-supplementary.pdf]

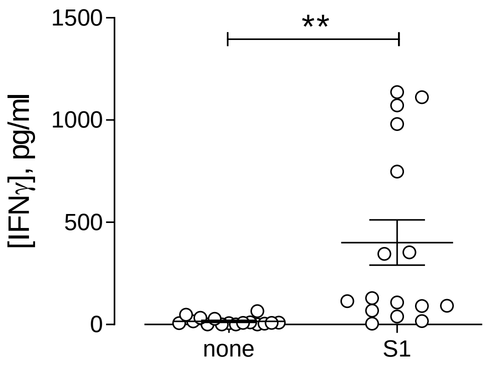

Figure S1. Monocyte derived macrophages (MDM) were left untreated (none) or incubated for 24 h with 5 nM spike S1 protein (S1). IFN-γ released in the medium were quantified with an ELISA assay, as described in Methods. Data are means ± SEM of sixteen independent experiments (single dots). **$p < 0.01$ vs. none with paired Student's t test.
